# Discovery of a novel rumen methanogen in the anaerobic fungal culture and its distribution in the rumen as revealed by real-time PCR

**DOI:** 10.1186/1471-2180-14-104

**Published:** 2014-04-23

**Authors:** Wei Jin, Yan Fen Cheng, Sheng Yong Mao, Wei Yun Zhu

**Affiliations:** 1 Laboratory of Gastrointestinal Microbiology, College of Animal Science and Technology, Nanjing Agricultural University, 210095 Nanjing, China

## Abstract

**Background:**

The novel archaea belonging to Rumen Cluster C (RCC), which may play an important role in methane production in the rumen have received increased attention. However, the present information on RCC in the rumen is limited by the unsuccessful isolation of axenic pure RCC from the rumen. In the present study, RCC grown in anaerobic fungal subcultures was identified by the molecular and culture methods.

**Results:**

A novel RCC species existing in the fungal subcultures was identified and demonstrated by the 16S rRNA gene clone library. Interestingly, the novel RCC species survived in the fungal cultures over all the subculture transferring, even in the 62^nd^ subculture, in contrast to the other methanogens, which disappeared during subcultures. Further work showed that subculture transfer frequency significantly affected the relative abundance of the novel RCC species in the fungal subcultures. The five-day and seven-day transfer frequencies increased the relative abundance of the RCC species (*P*<0.05). In addition, quantitative real-time PCR revealed that high concentrate diets did not affect the abundance of archaea, but numerically reduced the abundance of the novel RCC species in the rumen. In addition, the relative abundance of the RCC species was numerically higher in the rumen liquid fraction than in the rumen epithelium and solid fractions. Finally, a purified fungal culture containing the RCC species was successfully obtained. PCR and sequencing analysis showed that the novel RCC species contained a *mcr*A gene, which is known to play a crucial role in methanogenesis, and thus could be identified as a methanogen.

**Conclusion:**

In this study, a novel RCC species was identified as a methanogen and closely associated with anaerobic fungi. This novel approach by using co-culture with anaerobic fungi may provide a feasible way to culture and investigate not yet identified methanogens.

## Background

Methanogen diversity has been widely investigated across a range of ruminants by using clone library sequence approaches and many unknown methanogen 16S rRNA sequences have been uncovered. Tajima et al. [1] investigated the diversity of bovine rumen fluid using two different archaea-specific primer sets, and for the first time reported the existence of a novel cluster of uncultured archaeal sequences which were distantly associated with *Thermoplasma*. However, the authors concluded that these novel sequences were likely from transient microbiota contaminating the animal feed, probably scavenging in an ecological niche in the rumen. Wright et al. [2] was the first to verify that these novel *Thermoplasma*-affiliated sequences were derived from the rumen when they investigated the diversity of rumen methanogens from sheep. The authors suggested a new order of methanogens for these novel sequences in the new cluster. The same authors [3] further found that over 80% of the total methanogen clones (63 of 78 clones) from the rumen of Merino sheep in Australia were 72–75% similar to *Thermoplasmaacidophilum* and *Thermoplasmavolcanium*. They [4] also found that about 50% of the total clones from methanogen 16S rRNA gene library of potato-fed feedlot cattle were present in the new cluster, and 38% for corn-fed feedlot cattle. Huang et al. [5] found that *Thermoplasmatales*-affiliated sequences dominated in the yak and cattle methanogen clone libraries, accounting for 80.9% and 62.9% of the sequences in the two libraries, respectively. Our previous study [6] on the diversity of methanogens in the rumen of Jinnan cattle showed that *Thermoplasmatales*-affiliated sequences were widely distributed in the rumen epithelium, rumen solid and fluid fractions. In addition, ruminant-derived sequences in this new cluster were also found in other studies [4,7-12]. Based on the analysis of the global data set, Janssenand Kirs [13] placed the majority (92.3%) of rumen archaea detected in total rumen contents into three genus-level groups: *Methanobrevibacter* (61.6%), *Methanomicrobium*(14.9%), and a large group of uncultured rumen archaea affiliated with *Thermoplasmatales* (15.8%), and named the uncultured archaea group in the rumen, for the first time, as Rumen Cluster C (RCC). Using RCC specific DGGE, clone library analysis and quantitative real-time PCR, Jeyanathan et al. [11] investigated the composition of archaeal communities in the rumens of farmed sheep, cattle and red deer, and found that the 16S rRNA gene sequences of RCC were highly diverse and made up an average of 26.5% of the total archaea.

To date, the RCC has been found in many ruminants, including cattle [1,4,6-8,11], sheep [2,5,11], goats [9,12], water buffalo [10], and red deer [11]. Further the proportion of RCC within the total methanogen populations is high (up to 80%) [11,13]. However, most of these studies have been conducted using sequencing-based culture-independent molecular methods. The role of RCC in the rumen remains unclear in the absence of cultivated isolates. Further, although RCC has been labeled as a group of methanogens, there is little evidence to support that the RCC is methanogen [13].

Recently, Poulsen et al. [8] investigated the impact of rapeseed oil on the abundance of rumen microorganisms and their gene expression by metatranscriptomics, and found that methylamines might be the substrates for RCC. They further verified this by in vitro experiment which was composed of adding trimethylamine (TMA) to bovine rumen fluids and incubating for 24 hours. The results showed that methane production increased 22%, accompanied by a three fold increase for the abundance of RCC. Moreover, the recently reported *Methanomassiliicoccus luminyensis* from human feces, which was clustered within RCC clade in our present study, could use hydrogen to reduce methanol to methane [14]. Borrel et al. [15] published the genome sequence of another RCC related isolate (*Candidatus Methanomethylophilus alvus*) from human gut and reported this isolate contains genes needed for methylotrophic methanogenesis from methanol and methylamines. Padmanabha et al. [16] reported that a chicken gut isolate (*Methanoplasma gallocaecorum* strain DOK-1) belonging to RCC clade could strictly use hydrogen to reduce both methylamines and methanol to methane. In agreement with Wright et al. [2] suggesting a new order, Paul et al. [17] strongly proposed that these unclassified *Thermoplasmatales* sequences (as referred as RCC and its phylogenetic relatives) represents the seventh order of methanogenic archaea, based on the comparative phylogenetic analysis of the 16S rRNA genes and *mcr*A gene sequences, together with the enriched cultures from the higher termites and millipedes and the recently reported isolate *M. luminyensis*. Thus, the methanogenic archaeon in this order are widely distributed in marine habitat, soil, and in the intestinal tracts of termites and mammals.

Although the exact contribution of RCC to rumen methane production still remains unclear, they possibly play an important role in the methanogenesis, due to their high percentage in the rumen methanogen population [11,13]. Therefore, the cultivation and isolation of these unique RCCs from rumen has become increasingly important for understanding the role of RCC in the rumen. However, many attempts have been made, but the isolation of anoxic pure RCC from the rumen still remains unsuccessful.

Our previous studies showed that some rumen methanogens were indigenously associated with anaerobic fungi, which could result in a strong degradation of lignocellulosic materials [18,19]. Through an approach using a co-culture derived from a mixed-culture, our study further found that a novel species belonging to RCC grew in the anaerobic fungal subcultures. Therefore, the present study aimed to identify this novel species and investigate its features in the anaerobic fungal cultures. PCR specific primers were designed to monitor the novel RCC species growing in the fungal cultures and its distribution in the rumen. To better understand the novel RCC species, purification was also conducted.

## Results

### Presence of methanogens in the anaerobic fungal subcultures

**Figure 1:**
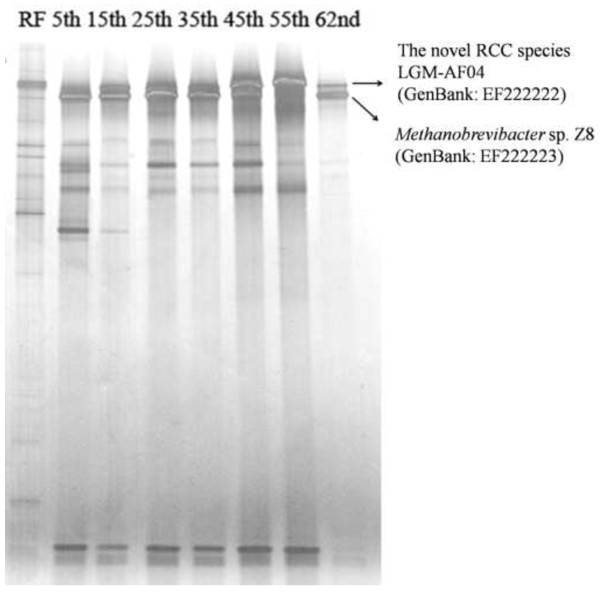
**DGGE profiles of methanogens in the mixed cultures.** RF, rumen fluid; 5^th^, the fifth subcultures; 15^th^, the fifteenth subcultures; 25^th^, the twenty-fifth subcultures; 35^th^, the thirty-fifth subcultures; 45^th^, the forty-fifth subcultures; 55^th^, the fifty-fifth subcultures; 62^nd^, the sixty-second subcultures; RCC: rumen cluster C.

**Figure 2:**
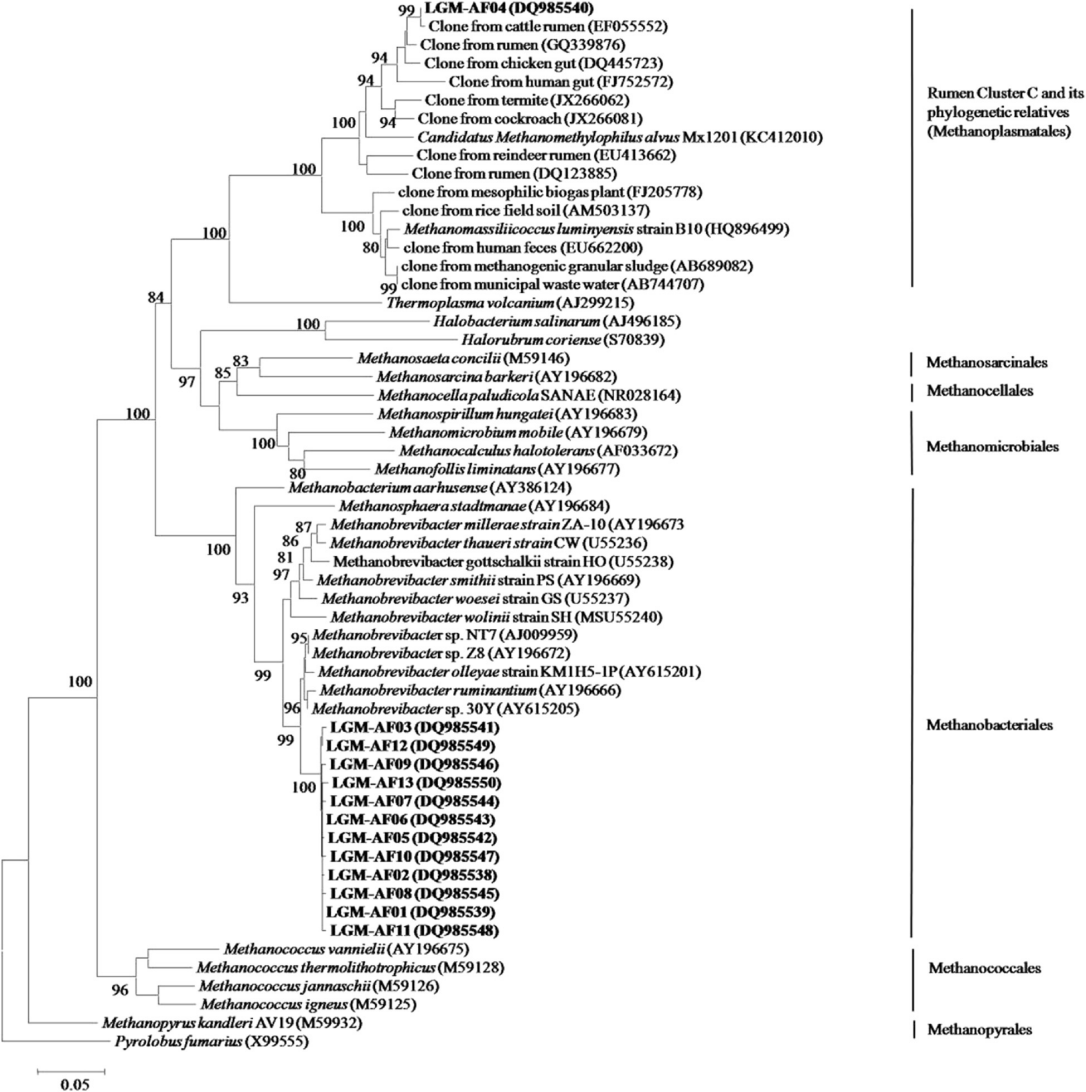
**Phylogenetic analysis of 13 phylotypes of methanogens from the 25th anaerobic fungal subculture.** The sequences determined in this study are marked in bold type. Accession numbers are given in parentheses. The root was determined by using *Pyrolobus fumarius* (× 9555) as outgroup. The topology of the tree was estimated by bootstraps, based on 1000 replications. Bootstrap values greater than 80% are shown on the internal nodes.

The methanogen diversity in the fungal cultures during transfers was shown in DGGE in Figure 1. As the consecutive transfer proceeded there was a reduction in the diversity of methanogens, resulting in only two strong bands on the gel of the 62^nd^ subcultures. In order to understand the composition of the methanogens in the enriched mixed cultures, a clone library targeting the 16S rRNA gene was constructed for the methanogens in the 25^th^ subcultures. A total of 66 clones were examined by riboprint analysis, and 13 phylotypes were revealed (Table 1). Two of these 13 phylotypes, represented by two clones, were 97.5%, 97.7% similar to *Methanobrevibacter* sp. 30Y, respectively. Ten phylotypes, represented by 62 clones, were 97.4% to 97.8% similar to *Methanobrevibacter* sp. Z8. One phylotype (LGM-AF04), represented by two clones, was 93.0% similar to *Ca. M. alvus* M × 1201. As shown in Figure 2, 12 of the 13 phylotypes were clustered into the “RO” cluster of the genus *Methanobrevibacter*. The phylotype LGM-AF04 was clustered with sequences representing RCC.

**Table 1:** Methanogen 16S rRNA gene clones from the 25th anaerobic fungal subculture

**16S rRNA phylotype**	**No. of clones**	**Size (bp)**	**GenBank accession number**	**Nearest valid taxon**	**Sequence identity (%)**
LGM-AF01	51	1260	DQ985539	*Methanobrevibacter*sp. Z8	97.8
LGM-AF02	1	1260	DQ985538	*Methanobrevibacter*sp. Z8	97.6
LGM-AF03	1	1260	DQ985541	*Methanobrevibacter*sp. 30Y	97.5
LGM-AF04	2	1256	DQ985540	*Candidatus Methanomethylophilus alvus* Mx1201	93.0
LGM-AF05	2	1260	DQ985542	*Methanobrevibacter*sp. Z8	97.7
LGM-AF06	1	1260	DQ985543	*Methanobrevibacter*sp. Z8	97.5
LGM-AF07	1	1260	DQ985544	*Methanobrevibacter*sp. Z8	97.6
LGM-AF08	2	1260	DQ985545	*Methanobrevibacter*sp. Z8	97.5
LGM-AF09	1	1260	DQ985546	*Methanobrevibacter*sp. Z8	97.6
LGM-AF10	1	1260	DQ985547	*Methanobrevibacter*sp. Z8	97.5
LGM-AF11	1	1260	DQ985548	*Methanobrevibacter*sp. Z8	97.5
LGM-AF12	1	1260	DQ985549	*Methanobrevibacter*sp. 30Y	97.7
LGM-AF13	1	1260	DQ985550	*Methanobrevibacter*sp. Z8	97.4

A total of 66 clones were examined.

Further, in order to understand the methanogens which survived in the long-term transferred fungal subcultures, the two strong bands from the 62^nd^ subcultures were excised from the DGGE gel for further cloning. Five clones generated from each band were sequenced and showed to be identical. One band had its sequence (EF222222) 99% similar to LGM-AF04, and the other had its sequence (EF222223) 98% similar to *Methanobrevibacter* sp. Z8.

### Transfer frequency affects the abundance of the novel RCC species in the fungal subcultures

**Figure 3:**
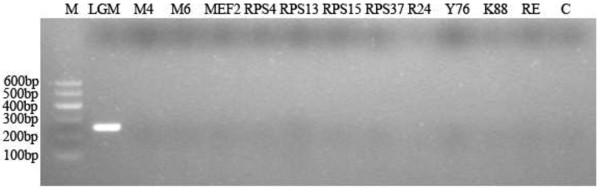
**Detection of the PCR specific primers for the novel RCC species.** M, DNA marker; LGM, the novel RCC clone; M4, *Methanobacterium beijingense* like strain; M6, *Methanobacterium formicicum* like strain; MEF2, *Methanobrevibacter smithii* like strain; RPS4/RPS15, *Methanoculleus* sp. like strain; RPS13/RPS37, *Methanosarcina mazei* like strain; R24, *Methanomicrobium mobile* clone; Y76, *Methanosphaera stadtmanii* clone; K88, *E. coli* K88; RE, *E. coli* isolated from rumen digesta; C, PCR control.

To monitor the abundance of the novel RCC species, PCR specific primers (LGM178f/434r) to this novel RCC were designed. BLAST searches of the primer sequences showed homology to sequences within the novel RCC species only. Their specificity was further confirmed by running PCR, and results showed that the primers only targeted the novel RCC species, and did not target other methanogen isolates or clones, or bacteria species tested in this study (Figure 3).

**Figure 4:**
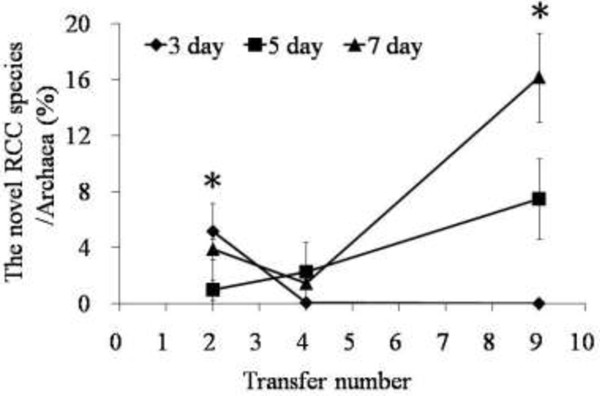
**The relative abundance of the novel RCC species in the anaerobic fungal cultures transferred with three transfer frequencies.** Fungal cultures were transferred every 3, 5, and 7 days, and the samples were collected at the 2^nd^, 4^th^, and 9^th^ subcultures. The data was calculated (16S rRNA gene number of the novel RCC species/Archaea) and shown with mean ± SD, n = 3.

The effects of the transfer frequency on the abundance of the novel RCC species in the anaerobic fungal subculture were investigated using the specific primers. The results showed that, as the transfer proceeded, the16S rRNA gene copy numbers of the novel RCC species significantly increased in the mixed cultures with the five-day transfer frequency and the seven-day transfer frequency (*P*<0.05), while it decreased in the three-day subcultures (Figure 4). This finding suggested that low transfer frequency might benefit the enrichment of the novel RCC species in the mixed cultures.

### Distribution of the novel RCC species in the rumen

The distribution of the novel RCC species in the rumen epithelium, in the liquid and solid fractions of goats fed with diets of different concentrate levels is shown in Table 2. The16S rRNA gene copy numbers of the novel RCC species in the rumen epithelium, the liquid and solid fraction ranged from 0.50 to 2.56, 14.44 to 93.45 and 50.30 to76.09 (×10^6^per cm^2^, ml or g), respectively. The total archaea ranged from16.34 to 36.68, 162.69 to 248.93 and 1385.19 to 2079.26 (×10^6^ per cm^2^, ml or g), respectively. The abundance of the novel RCC species in the rumen of goats fed low concentrate diet was numerically higher than that of goats fed high concentrate diet. But, the abundance of the total archaea was not affected by the high concentrate feeding. The relative abundance of the novel RCC species within total archaea (12.01 ± 6.35% to 56.47 ± 30.84%) in the liquid fraction was numerically higher than in the other two fractions (1.56 ± 0.49% to 29.10 ± 35.99% and 2.68 ± 2.08% to 5.71 ± 2.07%) in each diet group.

**Table 2:** The 16S rRNA copy numbers of the total Archaea and the novel RCC species in the rumen as quantified by real-time PCR

Level of concentrate inclusion*	**Archaea**			**The novel RCC species**			**The novel RCC species/Archaea**		
	**Epithelium × 10**^**6**^ **cm**^**2**^	**Liquid × 10**^**6**^ **ml**	**Solid × 10**^**6**^ **g**	**Epithelium × 10**^**6**^ **cm**^**2**^	**Liquid × 10**^**6**^ **ml**	**Solid × 10**^**6**^ **g**	**Epithelium %**	**Liquid %**	**Solid %**
High (65%)	33.25	133.94	2079.26	0.50^a^	14.44	50.30	1.56	12.01	2.85
Medium (40%)	36.68	248.93	1857.66	0.66^a^	30.97	38.46	12.90	19.06	5.71
Low (0%)	16.34	162.69	1385.19	2.56^b^	93.45	76.04	29.10	56.47	2.68
SEM	6.22	35.73	285.15	0.40	16.56	10.73	7.98	9.23	0.78
P-value	0.413	0.450	0.661	0.034	0.106	0.393	0.421	0.086	0.219

^a, b, c^, means with different letters in the same column are different *P* < 0.05; n = 3.

^*^, The pH value of rumen content, 5.60 ± 0.11 (High); 5.79 ± 0.15 (Medium); 6.17 ± 0.25 (Low).

### Purification of the novel RCC species with anaerobic fungus

One fungal culture containing the novel RCC species was obtained after purification with trimethylamine to support the novel RCC and with Lumazine to inhibit the growth of *Methanobrevibacter* sp. The anaerobic fungus was identified as belonging to *Piromyces* sp. as revealed by morphological examination (monocentricthallus; spherical or oval sporangium with filamentous rhizoids; uniflagellate zoospores).

The sequencing results showed only one 16S rRNA gene sequence from the total DNA extracted from the supernatant of the fungal culture, and this sequence was 100% identical to LGM-AF04 (DQ985540) and 99% to the clone from Jinnan cattle rumen (EF055552). Further confirmation was also performed by sequencing the *mcr*A gene coding the alpha subunit of the methyl-coenzyme M reductase that plays a crucial role in the methanogenesis, and the results showed that only one *mcr*A gene sequence (GenBank: KC859622) was present. These findings strongly supported the hypothesis that the novel RCC species in the co-culture with rumen anaerobic fungi is a methanogen.

## Discussion

In this study, a novel RCC species was found growing in the anaerobic fungal subcultures. Many studies have shown that a large group of RCC inhabited the rumen of a variety of ruminant species on various diets [1,2,4-11]. Thus, the RCC species grown in the anaerobic fungal cultures in the present study just represented a small group of the total RCC. It has been proposed that the RCC in the rumen and its relatives in other environments could constitute the seventh order of the methanogens (*Methanoplasmatales*) [17]. Methanogens within this new methanogenic order distantly related to the *Thermoplasmatales*, have been shown to be present in various environments, including marine habitats, soil, and also the intestinal tracts of termites and mammals, suggesting their ubiquitous in various environments. The whole order was proposed to form three big clusters, *Ca. M. alvus* Cluster, *M. luminyensis* Cluster and Lake Pavin Cluster [15]. The novel RCC species in the present study was grouped in the *Ca. M. alvus* Cluster. The present study reported the first account for RCC species grown in the fungal cultures from the goat rumen. Nevertheless this single species may not represent the whole RCC community in the rumen. Therefore, further research is needed to uncover this community and its features in the rumen.

Interestingly, this novel species could survive in the long-term transferred fungal subcultures (even in the 62^nd^ subcultures). Thus, there must be an underlying mechanism supporting the growth of this novel RCC species in the fungal subcultures. A similar phenomenon for protozoa was reported by Irbis and Ushida [20]. When testing a single protozoa cell for the 16S rRNA gene sequences of archaea, they found that the cultured rumen protozoa *Isotricha intestinalis* and *Ophryoscolex purkynjei* from goats carried *Thermoplasma* sp. related sequences (GenBank: AB189868, 99% similarity to LGM-AF04). Recent studies showed that methanogens belonging to this group [8,14-17] could strictly use hydrogen to reduce methanol and methylamines to methane. It is well known that both anaerobic fungi and protozoa could produce hydrogen, which is one of the substrates for methanogens [19,21]. This may make it possible for anaerobic fungi to provide RCC species with hydrogen. Methanol and methylamines could be derived from the microbial degradation of pectin, betaine, and choline from diets in the rumen [22]. Ametaj et al. [23] demonstrated that there were methanol and methylamines in the rumen fluid of lactating dairy cows fed graded amounts of barley grain. In this study, the medium for the co-culture of anaerobic fungi and methanogens contained rice straw and clarified rumen fluid. Anaerobic fungi could degrade the pectin of rice straw by pectinolytic enzymes [24,25], accompanying the release of methanol. Thus, it is possible that the novel RCC species obtained a certain amount of TMA and methanol from the rumen fluid and the degradation of rice straw.

When methanol was used to enrich RCC in the fungal cultures, *Methanosphaera* sp. was obtained instead of RCC species (unpublished), which implied that *Methanosphaera* sp. may compete for the same substrate (methanol) with RCC. In addition to the competition for the available substrates, there might be other underlying mechanisms enabling the novel RCC species to survive in the in vitro and *in vivo* niches. Apparently, further research is necessary to reveal the underlying mechanisms.

The novel RCC exhibited apparent enrichment with less frequent transfer, with relatively higher proportion in 7 day transfer culture than in 3 d or 5d transfer cultures (Figure 4). In our previous study, Cheng et al. [18] investigated the effects of transfer frequencies on the diversity of anaerobic fungi and methanogens in the enriched mixed cultures. They found that anaerobic fungal diversity was related to transfer frequencies and appeared to be simplified as transfer proceeded. In contrast, the methanogen population generally remained diverse, regardless of the transfer frequencies. Thus, the survival and the shift of the abundance of the novel RCC species in fungal cultures might be related to the changes of the composition of the anaerobic fungal community. On the other hand, it seems that the RCC grew slowly in the in vitro culture, while the *Methanobrevibacter* tended to grow more rapidly. Thus longer incubation interval between transfers would allow the RCC populations to increase while the *Methanobrevibacter* populations were declining. Therefore, the approach using long incubation intervals would allow the enrichment of the novel RCC. However, how much the transfer frequency effect may be due to the specific co-culture with an anaerobic fungus remains an open question.

The present study quantified the abundance of the novel RCC species and the total archaea in the rumen. It seems that the abundance of the novel RCC species was also affected by the diet composition, with the value in the rumen of goats fed low concentrate diet numerically higher than that of goats fed high concentrate diet (Table 2). But the abundance of the total archaea seems not affected by the levels of concentrate in the diets (Table 2). Similarly, Hook et al. [26] reported that high-concentrate feeding did not affect the density of the total rumen methanogens, but they found that high-concentrate feeding mitigated the methane production and altered the methanogen diversity and community structure. They also suggested that pH sensitive methanogens might be lost when the rumen pH decreased. It was possible that the novel RCC species was sensitive to low pH caused by high-concentrate feeding. It is also possible that some unaffected methanogens occupied the vacated niche of the novel RCC species in the rumen of goats fed with high-concentrate diet. Nevertheless, it is still unclear whether all the RCC populations are sensitive to the high-concentrate feeding and/or low pH.

The present study provided the first estimation of this RCC species distribution in the rumen. The abundance of the novel RCC species was different in the rumen epithelium, rumen liquid and solid fractions (Table 2). The relative abundance of the novel RCC species as indicated by its proportion within total archaea populations in their respective fraction was higher in liquid fraction as compared to epithelium and solid fraction. Previous study suggested that it was difficult to detach all of the microbes associated with the solid fraction [27], thus the abundance of RCC and archaea in this fraction may be grossly underrepresented. Our previous study [6] showed that the composition of the methanogens were different in the rumen epithelium, solid and liquid fractions of Jinnan cattle, especially for the unidentified archaea. We compared these unidentified archaeal sequences with RCC sequences (GenBank: AY351437, AY351466, DQ985540) in this study and found that 6.3% of the total clones in the liquid fraction was clustered within RCC clade, and 17.0% in the solid, 19.9% in the epithelium. The clones (GenBank: EF055552, 99%; EF055553, 98%; EF055554, 98%; EF055555, 98%; EF055556, 97%) that were most similar to the novel RCC species were from the rumen epithelium fraction. Moreover, Gu et al. [9] reported that 22.7% of the clones in the goat rumen fluid library belonged to the *Thermoplasmatales* family (as referred as RCC), and 63.2% in the rumen solid library; however, no clones were > 95% similar to the novel RCC species. In this study, the relative density of the novel RCC species was numerically higher in the rumen liquid fraction (12.01 ± 6.35% to 56.47 ± 30.84%) than in the other two fractions (1.56 ± 0.49% to 29.10 ± 35.99% and 2.68 ± 2.08% to 5.71 ± 2.07%), which might be due to the specific characteristics of the novel RCC species. In the rumen, liquid, solid and epithelium fractions have different turnover rates. Janssen and Kirs [13] proposed that the methanogens associated with different rumen fractions could be expected to have different growth rates since they would be removed from the rumen at different rates. Thus, the novel RCC species might have a relatively higher growth rate than other RCCs in the rumen liquid fraction.

In the present study, the novel RCC species was co-isolated with anaerobic fungus. Most recently, a tri-culture with a RCC member, a *Clostridium* sp. and a *Bacteroides* sp. was enriched from bovine rumen (Personal communication by Dr. Chris McSweeney, CSIRO, Australia). Further attempts to obtain pure RCC species were made but unsuccessful. It seems that there is a close relationship between the novel RCC species and anaerobic fungus. Two isolates (*Ca. M. alvus* Mx1201 [15] and *M. luminyensis*[14]) related to RCC had been obtained from human feces. Most recently, another RCC related isolate *M. gallocaecorum* strain DOK-1 [16] from chicken gut was reported. The three isolates exhibited similar nutritional requirements for methyl-group chemicals.

In the current study, the phylogenetic analysis showed that the novel RCC species were clustered into the same clade with *Ca. M. alvus* Mx1201 (Figure 2). However, the 16S rRNA gene sequence of the novel RCC species showed 93% similarity to *Ca. M.alvus*Mx1201 (GenBank: KC412010), and 87% to *M. luminyensis* (GenBank: HQ896499). The *mcr*A gene sequences of the novel RCC species (GenBank: KC859622) showed 84% similarity to *Ca. M. alvus* Mx1201 (GenBank: KC412011), and 78% to *M. luminyensis* (GenBank: HQ896500). Thereby, though clustered into the RCC clade, the novel RCC species in this study were phylogenetically distant with the two human isolates, the recently reported RCC isolates, suggesting that the new order for RCC and its relatives may be highly diverse.

## Conclusions

A novel RCC species was found surviving in the long-term transferred anaerobic fungal subcultures and closely associated with anaerobic fungi. The results verified that the quantification of the novel RCC species *in vivo* and in vitro is possible by real-time PCR using its specific primers. The relative abundance of the novel RCC species in the anaerobic fungal subcultures was affected by the transfer frequencies, with the seven day transfer frequency suitable for its enrichment. The high concentrate feeding did not affect the abundance of the total archaea population, but numerically reduced the abundance of the novel RCC species in the goat rumen. The relative abundance of the novel RCC species was numerically higher in the rumen liquid fraction than in the epithelium and solid fractions. A novel RCC species was co-isolated with an anaerobic fungus, and was identified as being a methanogen. The finding in the present study may help to culture and investigate the unknown methanogens in the rumen.

## Methods

### Ethics

All of the management, ethical and experimental procedures were conducted according to the protocols approved by the Animal Care and Use Committee of Nanjing Agricultural University, 1999.

### Animals and diets

Nine 3 year-old ruminally fistulated castrated male goats (Haimen goat) with weight at 29 ± 2 kg were kept on our university farm (Nanjing). The goats were randomly assigned to three diet groups (High concentrate diet, 64%: n = 3; Medium concentrate diet, 40%: n = 3; Low concentrate diet, 0%: n = 3). The experiment lasted for 22 days. The animals were maintained in individual pens with free access to water and fed twice daily at 0800 and 2000 hours. The diets contained mainly leymus chinensis, alfalfa, corn meal, wheat meal and soybean, with the ingredients and nutrient composition of the diet reported in our previous study [28]. The diets were offered for *ad libitum* intake to allow approximately 5% feed refusals.

On the day of sampling, the nine goats were slaughtered six hours after the morning feeding. Rumen fluid, rumen solid, and rumen epithelium samples were collected, respectively, according to the method described in our previous study [6]. Five pieces (3 cm × 3 cm per piece) of rumen wall were cut from the rumen of each goat. At the same time, microorganisms on the rumen epithelium were collected by scraping with glass slides. The rumen contents were divided into rumen fluid and solid fractions by squeezing through two layers of cheesecloth and centrifugation at 800 × g for 15 min at 4°C. All samples were stored at −70°C.

### Establishment and maintenance of the mixed-cultures of anaerobic fungi and methanogens

The mixed cultures of anaerobic fungi and methanogens were enriched from rumen content according to our previous study [29]. Rumen content was collected into pre-warmed thermos flasks from three rumen fistulated goats (Haimen goat) fed with Leymus Chinensis and immediately transported to the laboratory. The rumen content was homogenized prior to being squeezed through two layers of cheesecloth under anaerobic conditions. The resultant rumen liquid (5 ml) was placed into a CO_2_ gassed serum bottle with 45 ml of anaerobic diluting solution [30]. Three 10 ml aliquots were removed from the bottle and inoculated into three pre-warmed bottles (39°C) containing 90 ml of growth medium (Mixed-cultures). The mixed-cultures were incubated at 39°C in the incubator (PYX-DHS-50 × 65, Shanghai, China) without shaking and transferred every 3–4 days. In this study, the mixed cultures were transferred more than 62 times. A 7 ml portion of the culture supernatant from the 5^th^, 15^th^, 25^th^, 35^th^, 45^th^, 55^th^, and 62^nd^ subcultures and 1.5 ml of the goat rumen content were collected for DNA extraction.

Orpin’s medium [31] was prepared by boiling the mixture for 5 min prior to pumping with CO_2_ to remove O_2_. After 2–3 h gassing with CO_2_, the medium was then dispensed into 160 ml serum bottles sealed with butyl rubber septa and aluminium crimp-seals (Bellco Glass Inc., Vineland, New Jersey, USA) in anaerobic condition. The growth medium composed of Orpin’s medium containing penicillin (1600 IU/ml) and streptomycin (2000 IU/ml) and 1% ground rice straw (1 mm) as the substrate. Throughout this study, the growth of methanogens relied on the anaerobic fungi in the co-cultures and no additional hydrogen was added.

Methane produced by the mixed cultures was detected by GC during transfer, and the presence of methanogens in the mixed cultures was also monitored by PCR-DGGE.

In our previous study, transfer frequency was conducted to investigate its effect on the diversity and activity of enriched ruminal cultures of anaerobic fungi and methanogens in the mixed cultures [18]. DNA samples extracted from our previous study [16] were further analyzed for the novel RCC survival in the present study. Briefly, the mixed cultures of anaerobic fungi and methanogens were subcultured with three transfer frequencies (three-day, five-day, seven-day), respectively, each with triplicates. A portion of 5 ml culture supernatants from each of the 2^nd^, 4^th^, and 9^th^ subcultures was collected for DNA extraction. In the present study, the DNA samples were quantified for the total archaea and the novel RCC species and the survival of this RCC was investigated.

### Purification of the novel RCC species from the mixed-cultures

Fungal colonies containing the novel RCC species were purified from the mixed culture, according to our previous study [19]. Briefly, an aliquot of 0.5 ml of 10^−1^ to 10^−3^ diluted mixed culture was inoculated into 5 ml media with agar in Hungate roll-tube and incubated at 39°C in the incubator (PYX-DHS-50 × 65, Shanghai, China) without shaking. When the single fungal colonies formed after 5 days, colonies were picked up and transferred to fresh medium with cellobiose as substrate. This procedure was repeated several times to ensure that the colonies on the roll-tube were uniform. The obtained cultures were then checked for methane production by GC to ensure the presence of methanogens. RCC-specific PCR described below was used to confirm the presence of the novel RCC species existed in the purified fungal cultures.

During the purification, trimethylamine (Sigma-Aldrich, St Louis, MO, USA) was added to support the growth of the novel RCC species with the final concentration at 0.06 mol/L or 0.02 mol/L. Lumazine (Sigma-Aldrich, St Louis, MO, USA) was used to inhibit the growth of *Methanobrevibacter* sp. in the mixed-culture with its final concentration at 0.025%.

In order to confirm only the novel RCC isolate in the purified fungal culture. PCR was performed with the DNA extracted from the purified fungal culture and the PCR products were directly sequenced without cloning. The PCR primers used to amplify the 16S rRNA gene were 86f/1340r (Table 3). The PCR reaction system (50 μl) contained 5 μl of 10 × reaction buffer without MgCl_2_, 0.2 μM of both primers, 200 μM of each dNTP, 2 mM of MgCl_2_, 4 units of Taq DNA polymerase and1 μl of template DNA. The amplification parameters were as follows: initial denaturation at 94°C for 3 min, then 35 cycles of 94°C for 30 s, 58°C for 30 s and 72°C for 90 s, and last extension at 72°C for 10 min. To test whether the novel RCC is a methanogen, its DNA was subjected for amplification of the *mcr*A gene using primers MLf/MLr (Table 3). The PCR reaction system (50 μl) contained 5 μl of 10 × reaction buffer without MgCl_2_, 0.2 μM of each primer, 200 μM of each dNTP, 2 mM MgCl_2_, 4 unit of Taq DNA polymerase, and 1 μl of template DNA. Amplification parameters were as follows: 95°C for 5 min, 35 cycles of 95°C for 30 s, 55°C for 30 s and72°C for 1 min, and a final extension of 72°C for 7 min.

**Table 3:** Primers used in this study to target 16S r RNA genes of total archaea and the novel RCC species, mcrA genes of methanogens

**Target**	**Primers**	**Sequence (5’-3’)**	**Annealing temp (°C)**	**References**
Archaea*	915f 1386r	GTGCTCCCCCGCCAATTCCT GCGGTGTGTGCAAGGAGC	59	[11]
Methanogen	86f	GCTCAGTAACACGTGG	56	[2]
	1340r	CGGTGTGTGCAAGGAG		
The novel RCC species*	178f	TGGGATCTGGAATGACCCATGG	56	This study
	434r	TGAGAAAAGCTAGAACAAATGTCCT		
Methanogen^※^	519f	CAGCCGCCGCGGTAA	57	[39]
	915r^#^	CGCCCGCCGCGCCCCGCGCCCGGCCCGCCGCCCCCG		
Methanogen (*mcr*A gene)	MLf	GGTGGTGTMGGATTCACACARTAYGCWACAGC	55	[40]
	MLr	TTCATTGCRTAGTTWGGRTAGTT		

*, For real-time PCR; ^※^, For DGGE; ^#^, Where the primer name is prefaced by ‘GC-’ in the text, a GC-clamp was added to the 5’.

-terminus of the primer. The GC-clamp sequence was CCCCGTGCTCCCCCGCCAATTCCT;.

### DNA extraction

DNA extraction for the rumen epithelium (0.1 g wet weight) samples was conducted using a QIAamp® DNA Stool Mini Kit (QIAGEN, Hilden, Germany). Prior to extraction, the samples were pretreated using the FastPrep®-24 Instrument (MP Biomedicals, South Florida, USA). Then, the procedure followed the kit instructions.

DNA extraction for the culture supernatant (5 ml), the rumen fluid (3 ml), and the solid samples (0.3 g wet weight) were conducted using the cetyltrimethylammonium bromide method [32]. Prior to extraction, all the samples were washed two or three times with PBS buffer.

The DNA extracts were dissolved in 100 μl TE buffer and DNA yield was quantified using a NanoDrop ND-1000 Spectrophotometer (Nyxor Biotech, Paris, France). The DNA extracts were diluted in ddH_2_O prior to PCR reactions and 1 μl of the diluted DNA solutions (*c*.10 ~ 20 ng) were used as templates.

### PCR-DGGE analysis of methanogen community in subcultures of the co-culture with anaerobic fungi

PCR-DGGE analysis of the methanogen community in co-culture with anaerobic fungi was conducted with primers 519f/915GCr (Table 3) according to the methods described in our previous study [12]. The PCR reaction system (50 μl) contained 0.2 μM of both primers, 240 μM of each dNTP, 1.5 mM of MgCl_2_ and 2.5 units of Taq DNA polymerase, 1 μl of template DNA. The amplification parameters were as follows: initial denaturation at 94°C for 4 min, then 35 cycles of 94°C for 30 s, 57°C for 40 s and 72°C for 40 s, and last extension at 72°C for 10 min.

DGGE was performed using a Dcode DGGE system (Bio-Rad, Hercules, USA) with 6% (w/v) polyacrylamide gels (acrylamide/N, N’-methylene bisacrylamide ratio, 37: 1 [w/w]) in 0.5 × TAE buffer. The denaturant gradient range of the gel was from 35% to 75%, in which 100% denaturant contained 7 mol · L^−1^ urea and 40% (v/v) formamide. The electrophoresis was initiated by pre-running for 10 min at 200 V and subsequently ran at 85 V for 16 h at 60°C. The gel was stained with AgNO_3_ and scanned using GS-800 scanner (Bio-Rad, Hercules, USA). The DGGE profile was analysed by Molecular Analyst 1.61 software (Bio-Rad, Hercules, USA). DGGE bands were excised from the gel and rinsed with ddH_2_O. The DNA of each band was eluted in sterile TE buffer by incubation for 12 h at 37°C, and served as the template for re-amplification with primers 519f/915r. The PCR products of re-amplification were cloned in *Escherichia coli* Top10 by using the pGEM-T Easy Vector System (Promega, Madison, WI, USA). The inserts were screened by running the DGGE gel, compared with the original samples, and then sequenced by Invitrogen BioTech (Shanghai, China). For each band, more than 10 clones were picked up and 5 of them were sequenced.

### Clone library construction for methanogens in the mixed-cultures and phylogenetic analysis

The 25^th^ mixed-subculture was used for construction of methanogen clone library using PCR primers 86f/1340r. The PCR reaction system and amplification parameters were described above. PCR product was purified using a PCR Clean-Up system (Promega, Madison, WI, USA) and cloned into *E. coli* strain TOP10 using the pGEM-T Easy vector (Promega, Madison, WI, USA). The plasmids were re-amplified by PCR using the primers and parameters described above. The PCR products were digested initially with restriction enzyme *Hae*III (Fermentas, Canada), according to the manufacturer’s specifications. Digested DNA fragments were separated on a 4% molecular screening agarose gel (Biowest, Spain) running at 100 V.

Restriction fragment length polymorphisms were grouped according to their riboprint pattern and compared to a riboprint database for identification [33]. In some cases, when two or more strains had the same *Hae*III riboprint, an additional digestion with *Alu* I, *Hpa* II and *Sau* 3A (Fermentas, Canada) were applied to further differentiate the closely related strains. All the riboprints that differed from one another were sequenced. Sequencing was performed by Invitrogen BioTech (Shanghai, China) and all the sequences were confirmed by using the Basic Local Alignment Search Tool (BLAST) in GenBank.

The phylotypes were designated by using the prefix LGM, followed by AF to indicate the origin of the clones, and a number to identify each phylotype. The GenBank accession numbers for these phylotype sequences range from DQ985538 to DQ985550.

The methanogen phylotypes generated above were subjected for phylogenetic analysis. The phylogenetic analysis included 16S rRNA gene sequences downloaded from GenBank and the sequences obtained in this study. *Pyrolobus fumarius* (×99555) was used as an outgroup. The phylogenetic software MEGA5.1 was used to calculate the sequence similarities and the evolutionary distances between the pairs of nucleotide sequences determined, using the Kimura two-parameter correction model [34]. A distance matrix tree was then constructed using the neighbor-joining method [35] and bootstrap resampled was conducted 1000 times [36].

### PCR primers designed for the detection of the novel RCC species

PCR primers were designed targeting the 16S rRNA gene. Multiple alignments of the 16S rRNA genes were used to identify specific regions of the novel RCC species using DNASTAR® software. The primers were then designed from multiple alignments of the 16S rRNA genes of 26 methanogenic archaea. These sequences were available in GenBank (DQ372975, DQ355967, DQ372972, DQ355968, DQ985540, AB301476, AF531178, AY196675, AY196679, AY196682, AY196684, AY386124, AY487204, M59126, U55240, AF033672, AF095276, AJ009959, AY196669, AY196672, AY196677, AY196683, AY615205, M59125, and M59128). The forward primer was (LGMf) 5’-TGGGATCTGGAATGACCCATGG (*E. coli* 16S rRNA gene: 178–199); the reverse primer was (LGMr) 5’-TGAGAAAAGCTAGAACAAATGTCCT (*E. coli* 16S rRNA gene: 410–434). The specific PCR primers (LGM178f/434r) would amplify approximately 250 bp products.

The primers were compared with the sequences available at NCBI via a BLAST search to ascertain primer specificity. PCR assays using this specific primer pair were also performed to ascertain the primer’s specificity with DNA from the novel RCC clone and the negative controls. A number of strains isolated from our previous work [37] and clones from our another work [6] were used as negative controls, and these included isolates *Methanobacterium beijingense* like strain, *Methanobacterium formicicum* like strain, *Methanobrevibacter smithii* like strain, *Methanoculleus* sp. like strain, *Methanosarcina mazei* like strain, and clones *Methanomicrobium mobile* and *Methanosphaera stadtmanii*, and bacterial species *E. coli* K88, and *E. coli* isolated from rumen digesta. The PCR reaction system (20 μl) contained 2 μl of 10 × reaction buffer without MgCl_2_, 1.5 mM MgCl_2_, 200 μM of each dNTP, 0.2 μM of each primer, 1.5 unit of Taq DNA polymerase, and 1 μl of template DNA. The amplification parameters were as follows: 5 min at 95°C; 30 cycles, 15 s at 95°C, 30 s at 56°C, 45 s at 72°C; 4 min at 72°C. Aliquots of 5 μl PCR products were analyzed by electrophoresis on 2% (w/v) agarose gel (Biowest, Spain).

### Real-time PCR quantification of the novel RCC species and the total methanogens

For real-time PCR quantification, plasmid DNA to be used as the PCR standards were obtained by PCR cloning using the primer sets of LGM 178f/434r for the novel RCC species described above and 915f/1386r for archaea (Table 3), respectively. Plasmids containing respective target DNA fragments were used as standard for the novel RCC species and the total archaea, respectively. The concentration of the plasmid was quantified by using a Qubit ds DNA HS Assay Kit (Invitrogen, Eugene, Oregon, USA) on a Qubit 2.0 Fluorometer (Invitrogen, Carlsbad, CA, USA). The copy number of each standard plasmid was calculated using the molecular weight of the nucleic acids and the length (in base pairs) of the cloned standard plasmid [38]. A 10-fold dilution series ranging from 10 to 10^9^ copies was prepared for each target. To assess the sensitivity and accuracy of assays, the quantification range was determined using the serial dilutions of standard plasmid as the template.

Real-time PCR was performed using an Applied Biosystems 7300 Real-Time PCR System (Applied Biosystems, California, USA). The reaction mixture (20 μl) consisted of 10 μl of SYBR Green Real-Time PCR Master Mix (Toyobo, Osaka, Japan), 0.2 μM of each primer, and 2 μl of the template DNA (DNA was diluted 1/100). The temperature program for the novel RCC species consisted of denaturation at 95°C for 5 min, followed by 40 cycles consisting of 95°C for 15 s, annealing at 56°C for 30 s, and extension at 72°C for 45 s. The temperature program for the archaea consisted of denaturation at 95°C for 2 min, followed by 40 cycles consisting of 95°C for 15 s, annealing and extension at 60°C for 1 m. Melting curve analysis was conducted over a range of 60 to 95°C to assess specificity of the amplification products. The 10-fold dilution series of the standard plasmid for the respective target was run along with the samples. Amplification of each sample was performed in triplicate. Quantification was based on standard curves obtained from the amplification profile of known concentrations of the standard plasmid for the respective target. The total numbers of methanogens per gram wet weight or ml or cm^2^ were determined using ABI SDS software (Applied Biosystems, Foster City, CA, USA) and according to dilution factor and volume of DNA extracts.

### Methane detection

Methane was detected by GC (Shimadzu, gas chromatograph GC-14 B, Japan) according to the method described in our previous study [19]: capillary column (Supelco, Column No. 41491-03B, US) temperature 80°C, vaporizer temperature 100°C, flame ionization detector temperature 120°C, carrier gas (N_2_) pressure 0.05 MPa, H_2_pressure 0.05 MPa and air pressure 0.05 MPa.

### Identification of anaerobic fungus

The anaerobic fungi in the cultures were examined by microscopy (Eclipse 80i, Nikon, Japan) with DAPI (4’, 6 diamidino-2-phylindole) staining according to our previous study [19]. An aliquot of 1 ml 3-day old culture was treated with 1 μl, 500 μg/ml DAPI (Sangon Biotech (Shanghai) co., Ltd., Shanghai, China), stored in dark room for 5 min, and then examined by fluorescence microscopy.

### Statistical analysis

All data were analyzed by Tukey’s analysis of one-way ANOVA of SPSS 18.0 (SPSS, Chicago, IL, USA) at a 95% significance level.

## Competing interests

The authors declare that they have no competing interests.

## Authors’ contributions

WJ isolated the co-culture of the novel RCC isolate with anaerobic fungus, performed DNA extraction and q-PCR, analyzed the data and drafted the manuscript. YFC enriched the fungal culture, constructed the clone library, designed PCR primers for the novel RCC, performed PCR-DGGE analysis and drafted the manuscript. SYM performed the animal experiment and provided critical discussions during revision. WYZ conceived this study, finalized the manuscript and revised the manuscript. All authors read and approved the final manuscript.
